# Prognostic Significance of a Scoring System Combining p16, Smoking, and Drinking Status in a Series of 131 Patients with Oropharyngeal Cancers

**DOI:** 10.1155/2021/8020826

**Published:** 2021-09-07

**Authors:** Cyril Bouland, Didier Dequanter, Jérôme R. Lechien, Charlotte Hanssens, Nicolas De Saint Aubain, Antoine Digonnet, Rokneddine Javadian, Antoine Yanni, Alexandra Rodriguez, Isabelle Loeb, Fabrice Journe, Sven Saussez

**Affiliations:** ^1^Department of Stomatology-Maxillofacial Surgery, CHU Saint Pierre, Université Libre de Bruxelles (ULB), Brussels, Belgium; ^2^Department of Otolaryngolology-Head & Neck Surgery, CHU Saint Pierre, Université Libre de Bruxelles (ULB), Brussels, Belgium; ^3^Department of Thoracic-Head & Neck Surgery, Centre Oscar Lambret, Lille, France; ^4^Laboratory of Human Anatomy and Experimental Oncology, Faculty of Medicine and Pharmacy, Research Institute for Health Sciences and Technology, University of Mons (UMONS), Mons, Belgium; ^5^Department of Pathology, Jules Bordet Institute, Université Libre de Bruxelles (ULB), Brussels, Belgium; ^6^Department of Head and Neck Surgery, Jules Bordet Institute, Université Libre de Bruxelles (ULB), Brussels, Belgium; ^7^Laboratory of Oncology and Experimental Surgery, Bordet Institute, Université Libre de Bruxelles (ULB), Brussels, Belgium

## Abstract

**Background:**

Tobacco and alcohol are two main risk factors associated with head and neck squamous cell carcinoma (HNSCC). Studies showed that human papillomavirus (HPV) plays a role in the etiology of this cancer. HPV-positive oropharyngeal squamous cell carcinoma (OSCC) patients present in general a better response to conventional therapy and better overall survival (OS). However, OSCC is a heterogeneous disease regarding treatment. This study aimed to identify more effective prognostic factors associated with a poor clinical outcome for OSCC patients to improve treatment selection.

**Materials and Methods:**

OSCC patients diagnosed between 2007 and 2017, in two Belgian hospitals, were included. Demographic and clinicopathologic data were extracted from medical records. HPV status was determined through p16 immunohistochemistry. Univariable and multivariable Cox proportional hazard regression analyses allowed to identify variables prognostic for OS and recurrence-free survival (RFS). Kaplan–Meier survival curves have been assessed for survival.

**Results:**

The study included 131 patients. Statistics showed that monotherapies were significantly associated with a shorter OS; p16 overexpression was significantly associated with a weak consumption of tobacco or alcohol, and a high p16 expression was significantly associated with both longer RFS and OS. The study validated that tobacco and alcohol consumption were significantly correlated with poorer RFS and poorer OS. Only p16 expression trended to be significant for RFS when compared to smoking and drinking habits, while p16 upregulation and alcohol use were both vital for OS indicating that p16 is an independent and significant prognostic factor in OSCC patients. Finally, a scoring system combining p16, tobacco, and alcohol status was defined and was significantly associated with longer RFS and longer OS for nonsmoker and nondrinker p16-positive OSCC patients.

**Conclusions:**

This study confirmed that the overexpression of the p16 protein could be viewed as a factor of good prognosis for RFS and OS of OSCC patients. The prognostic significance of a scoring system combining p16 expression, smoking, and drinking status was evaluated and concluded to be a more effective tool to determine therapeutic orientations based on the risk factors for better treatment relevance and survival.

## 1. Introduction

Head and neck squamous cell carcinoma (HNSCC) is, worldwide, the sixth common cancer responsible for approximately one half million cancer cases every year [1]. Tobacco and alcohol are the two main risk factors associated with the development of this cancer type. Nowadays, epidemiologic studies have revealed that high-risk human papillomavirus (HPV) infection is etiologically linked to HNSCC pathogenesis [2,3]. HPV-positive HNSCCs are predominantly found in the oropharyngeal regions with an occurrence of 25–47% [4]. They are now recognized as a distinct HNSCC entity due to their demographic, histologic, clinical, and molecular differences [5]. Recent clinical studies have highlighted that patients with HPV-positive oropharyngeal squamous cell carcinoma (OSCC) present a better response to conventional therapy and better overall survival (OS) compared to HPV-negative OSCC patients [6].

Nevertheless, HPV-positive HNSCC is a heterogeneous disease regarding its response to treatment. The selection of patients and therapy only based on HPV status should be further evaluated. This study aimed to identify prognostic factors associated with a poor outcome for OSCC patients to improve treatment selection for those patients.

## 2. Materials and Methods

### 2.1. Sample Collection and Characteristics

OSCC patients diagnosed between 2007 and 2017 in the departments of Head and Neck Surgery of two Belgian hospitals (Saint-Pierre Hospital and Jules Bordet Institute) were recruited. The following factors were assessed: tumor differentiation, invasion, and staging; smoking history; alcohol habits; HPV status; treatment type and response; recurrence-free survival (RFS); and OS. The patients with a minimum follow-up of 12 months were included. Demographic and clinicopathologic data were extracted from their medical records. Our study was accepted by two ethics committees (Saint-Pierre Hospital: B076201835031 and Jules Bordet Institute: CE2857).

### 2.2. Immunohistochemistry for p16

Formalin-fixed paraffin-embedded tissue was tested for high-risk HPV through p16 immunohistochemistry by using mouse monoclonal antibody (CINtec p16, clone E6H4, Ventana, Tucson, AZ, USA) and an automated immunostainer (BenchMark XT System, Ventana). The p16 expression was defined as positive if both the nucleus and the cytoplasm were stained in more than 70% of tumor cells with at least a moderate to strong intensity.

### 2.3. Statistical Analysis

Univariable and multivariable Cox proportional hazard regression analyses were used to identify variables prognostic for OS and RFS. Kaplan–Meier survival curves were assessed for OS and RFS.

### 2.4. Prognostic Score

A prognostic score combining p16 expression, tobacco, and alcohol was designed in order to improve the prognosis of OSCC patients. These three factors were each associated with both RFS and OS. We considered data for p16 expression (0, positive IHC and 1, negative), tobacco (0, no/weak use and 1, moderate/high), and alcohol (0, no/weak consumption and 1, moderate/high). Each level corresponds to the number of drinks per day. Weak equals ≤1 drink/day, moderate: 2-3 drinks/day, and high: ≥ 4drinks/day [7]. The pooled relative risk (RR) was 1.21 (95% CI: 1.10–1.33) for ≤1 drink/day and 5.24 (95% CI: 4.36–6.30) for ≥4 drinks/day. We rated tobacco consumption as weak (≤ 10 pack-years), moderate (11–20 packs-years, and high (>20 pack-years), considering that O'Sullivan et al. highlighted *a* >10 pack-years tobacco exposure being a strong adverse OS predictor [8]. The score ranges from 0 to 3. A score of 0 or 1 (score 0/1) was associated with a good prognosis, while a score of 2 or 3 (score 2/3) corresponded to a poor one.

## 3. Results

**Figure 1:**
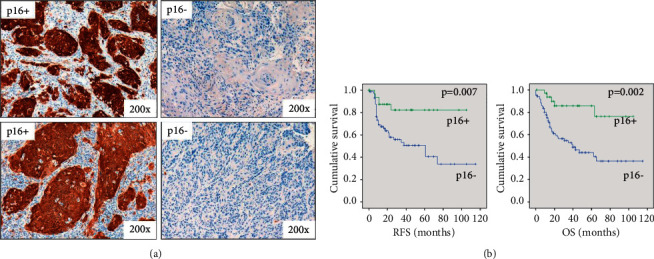
Evaluation of the p16 level in oropharyngeal tumors and association with survivals. (a) Assessment of p16 positivity in OSCC patients by immunohistochemistry. High p16 immunostaining corresponding to a transcriptionally active HPV infection (p16+). (b) Evaluation of RFS and OS regarding p16 status. Patients with p16+ tumors (*n* = 36) have a significant longer RFS (HR: 3.6, 95% CI: 1.4–9.3) and OS (HR: 4.3, 95% CI: 1.7–10.9) compared to p16− tumors (*n* = 88).

A total of 131 OSCC patients were included in the study. Among those, 88 patients were male (69%) and 43 were female, with a median age of 59 years old (ranging from 32 to 87) (Table 1). The majority of the patients were heavy smokers (moderate (*n* = 16) and high (*n* = 85)) and heavy drinkers (moderate (*n* = 21) and high (*n* = 71)). When evaluated for HPV status, 36/124 (29%) demonstrated high positivity for p16 expression (Figure 1(a)). Seven patients were not tested for HPV coinfection. The majority of the patients were treated by radiochemotherapy (37%) or primary surgery, followed by radiochemotherapy (24%) (Table 1).

Patients treated with monotherapies (radiotherapy or surgery alone) had a significantly shorter OS (HR: 1.9, 95% CI: 1.1–3.5, *p*=0.032, Cox regression), supporting the benefit brought by the multitherapy for OSCC patients.

Notwithstanding, p16 overexpression was significantly associated with a low tobacco or alcohol consumption (*p* < 0.001, Mann–Whitney test), suggesting that a population HPV infection is more active in cancer patients with a low smoking or drinking history.

Within the 131 patients group, high p16 expression, reflecting a transcriptionally active HPV infection in OSCC, was significantly associated with longer RFS (HR = 3.6, *p*=0.007, Cox regression) and longer OS (HR = 4.3, *p*=0.002) (Figure 1(b)). This demonstrated that p16 overexpression might be viewed as a strong marker of favorable prognosis in such cancer patients.

**Figure 2:**
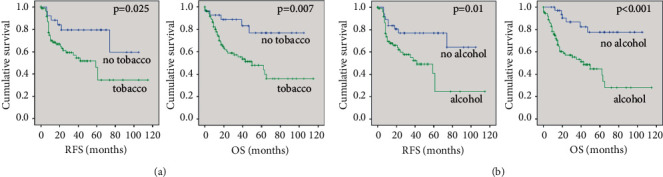
Survival and tobacco or alcohol habits in OSCC patients. (a) The prognosis for non- and light smokers (no tobacco, *n* = 28) significantly better than for moderate and high smokers (tobacco, *n* = 101) regarding to RFS (HR: 2.7, 95% CI: 1.1–6.5) and OS (HR: 3.6, 95% CI: 1.4–9.2). (b) The survivals for non- and light drinkers (no alcohol, *n* = 37) significantly longer than for moderate and high drinkers (alcohol, *n* = 92) regarding to RFS (HR: 2.7, 95% CI: 1.3–5.7) and OS (HR: 4.7, 95% CI: 2.0–11.2).

The study validated that tobacco and alcohol consumption, classical risk factors, were significantly associated with poorer RFS (HR: 2.7, *p*=0.025, and HR: 2.7, *p*=0.01, respectively) and OS (HR: 3.6, *p*=0.007, and HR: 4.7, *p* < 0.001, respectively) (Figure 2).

Multivariate analysis demonstrated that only p16 expression trended to be significant (*p*=0.05) for RFS when compared to smoking and drinking habits, while p16 expression upregulation (*p*=0.04) and alcohol use (*p*=0.04) were both significant for OS (Table 2), indicating that p16 expression is an independent and significant prognostic factor for OSCC patients.

**Figure 3:**
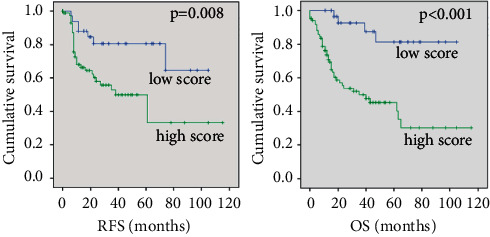
Evaluation of the prognostic performance of a score combining p16 and tobacco/alcohol habits in OSCC patients. Patients with a low score (p16+, no tobacco, no alcohol, *n* = 36) have a significantly longer RFS (HR: 3.1, 95% CI: 1.3–7.0) as well as a better OS (HR: 6.6, 95% CI: 2.3–18.4) than patients with a high score (p16−, tobacco, alcohol, *n* = 87).

The prognostic score highlighted that a score of 0/1 was significantly associated with longer RFS (HR: 3.1, *p*=0.008, Cox regression) and longer OS (HR: 6.6, *p* < 0.001) (Figure 3), supporting its use for the evaluation of OS in those patients.

## 4. Discussion

The results of the present study showed longer RFS and OS for HPV-positive OSCC patients, corroborating the current data in the literature [9,10]. Nevertheless, HPV-positive HNSCC is a heterogeneous disease regarding response to treatment. The selection of patients and therapies only based on HPV status has to be further evaluated. A first major drawback of HPV status as a biomarker for further treatment selection was reported by Ang et al. [11]. They demonstrated that 30% of HPV-positive patients showed an intermediate risk for survival when p16 expression was associated with tobacco consumption. In addition, Descamps et al. confirmed that the risk of death increased in HNSCC, especially in OSCC, when patients are exposed to tobacco and alcohol during their therapy, regardless of their HPV status [12]. Hence, the use of p16 expression, tobacco, and alcohol status, as risk factors, is not clearly defined to predict survival.

In the literature, many studies aimed to evaluate the biological importance and prognostic significance of p16 expression and selected specific clinical parameters for OSCC patients. As found in the present study, Laco et al. reported that OS of HPV-positive OSCC patients was significantly longer (median: 112 months, 95% CI: 54–112 months) than HPV-negative OSCC patients (median 17 months, 95% CI 12–39 months) (*p* < 0.001). Interestingly, in their study, smoking itself did not seem to be an important prognostic factor [13]. The improved prognosis observed for HPV-positive compared to HPV-negative squamous cell carcinomas patients was confirmed in a prospective trial [4]. Indeed, after a median follow-up of 39 months, patients with HPV-positive tumors had an improved OS (the 2-year OS was 95% (95% CI: 87–110%) vs. 62% (95% CI: 49–74%)). After adjustment for age, tumor stage, and performance status, patients with HPV-positive tumors had lower risks of progression (HR: 0.27, 95% CI: 0.10–0.75) and death (HR: 0.36, 95% CI: 0.15–0.85) than those with HPV-negative tumors. But, as demonstrated by Ang et al. [10], p16 expression does not seem to be the only key factor for survival of OSCC patients. Recently, Anantharaman et al. [14] reported, in a European multicentre study, that HPV 16 status, but not smoking status, has been found as an independent prognostic factor for survival. However, Beitler et al. [15] demonstrated that age, smoking, N3 disease, T4 disease, and a negative p16 expression were associated with the development of distant metastases in patients with squamous cell cancers of the oropharynx.

In the current study, the HPV diagnosis performed method was p16 IHC, alone. We considered p16 immunohistochemical positivity when at least 70% nuclear and cytoplasmic expression and at least moderate to strong intensity were observed, as recommended by the College of American Pathologists [16]. p16 is considered as a surrogate and a prognostic marker [17,18]. Indeed, DNA detection is not a sufficient standalone modality. Several HPV-specific testing modalities, with their pros and cons, exist [17]. In the case of neck fine-needle aspiration, it is considered as a compromise [17]. The detection of HPV oncogene E6/*E*7 transcripts is considered as the “gold standard” in tissue sample. HPV-driven carcinomas critically depend on the carcinogenic action of the HPV E6 and E7 oncogenes [17, 19]. Compared to p16 IHC, high-risk HPV RNA in situ hybridization presents the same sensitivity (97%) but a better specificity (93% vs. 82%) [20]. P16 immunostaining yields false-positive results in 5–20% of cases [21]. RT-PCR approaches and even RNAseq are great at detecting high-risk HPV E6/*E*7 mRNA but just are not practical for clinical application [17]. The detection of viral transcripts is laborious and may not be feasible in routine, especially for transcript detection from formalin-fixed paraffin-embedded (FFPE) specimens due to the reduced quality of RNA in this material. HNC samples are frequently stored and processed as FFPE tissue posing a challenge to the detection of HPV transcripts [19]. Furthermore, compared to other HPV-specific tests, p16 IHC is generally available, and technical costs are 2–16 times lower [22]. Notwithstanding, promising alternatives are emerging, such as liquid-based HPV testing of the supernatant generated in every single cervical lymph node FNA; high-risk HPV serology and circulating tumor cell and/or HPV DNA; computerized analysis of medical images, including cross-sectional radiology and digitized H&E pathology slides; and machine learning from clinical and pathologic features in the electronic medical record to diagnose HPV-positive OPSSC [17].

In the present study, to better take into consideration the different classical prognostic factors, a scoring system combining p16 expression, smoking, and drinking habits has been established. A significant correlation between the resulting score and both RFS and OS has been highlighted. Hence, nonsmokers, nondrinkers, and positive p16 expression patients had a better prognosis as well as patients with two of the three cited parameters (either nonsmokers, nondrinkers, or positive p16 expression). This score highlights the prognostic role of alcohol consumption that had not been evaluated previously. Several limitations can be listed in this study: the retrospective nature of the study and the small size of the cohort (131 patients), among those, only 36 were HPV-positive and are the major ones. Moreover, the majority of the HPV-positive patients were also heavy smokers and/or drinkers. On the other hand, the proposal to take into consideration p16 status, smoking, and drinking habits all together into a score is innovative from a clinical point of view while looking for additional prognostic and predictive tools. Moreover, these results suggest the necessity of complementary studies, analyzing other factors than the typical risk factors, integrating other clinical anatomy pathological factors to accurately identify individuals at risk of reduced outcomes in a good prognosis positive p16 expression OSCC patient's cohort.

## 5. Conclusions

This study confirmed that the overexpression of the p16 protein could be viewed as a good prognosis factor of RFS and OS for OSCC patients. The prognostic significance of a scoring system combining p16 expression, smoking, and drinking status was evaluated. The assessment of this score allows us to adopt a more effective tool to determine the therapeutic orientations based on the risk factors for a better treatment relevance and survival.

## Figures and Tables

**Table 1:** Characteristics of the OSCC patients (*n* = 131).

Parameters		*n*	Median	Range
Gender	Male	88		
	Female	43		
Age (years)		131	59	32–87
Differentiation	No	45		
	Moderate	36		
	High	36		
Invasion	No	4		
	Yes	121		
Stage	I	9		
	II	10		
	III	24		
	IV	85		
p16	No	88		
	Yes	36		
Tobacco	No	19		
	Weak	9		
	Moderate	16		
	High	85		
Alcohol	No	22		
	Weak	15		
	Moderate	21		
	High	71		
Treatment	Radio	24		
	Radiochemo	48		
	Surgery	12		
	Surgery + radiochemo	31		
	Surgery + radio	12		
Response	No	22		
	Yes	109		
RFS (months)		131	15	0–115
Recurrence		43		
OS (months)		131	19	0–115
Death		49		

**Table 2:** Multivariate analysis evaluating the correlation between p16 positivity in tumor, tobacco and alcohol consumption, and patient survivals (RFS and OS).

	*P* value	HR	95% CI
RFS			
p16	**0.05**	2.74	0.98–7.65
Tobacco	0.53	1.40	0.48–4.08
Alcohol	0.47	1.40	0.56–3.52
OS			
p16	**0.04**	3.15	1.03–9.64
Tobacco	0.67	1.28	0.41–3.98
Alcohol	**0.04**	2.87	1.04–7.95

Significant *p* values are highlighted in bold.

## Data Availability

The data used to support the findings of this study are available from the corresponding author upon request.
